# Explorative cost-effectiveness analysis of colorectal cancer recurrence detection with next-generation sequencing liquid biopsy in Spain, France, and Germany

**DOI:** 10.1177/17562848241248246

**Published:** 2024-05-10

**Authors:** Wendelin Schramm, Yasmin Hollenbenders, Maximilian Kurscheidt

**Affiliations:** GECKO Institute for Medicine, Informatics and Economics, Hochschule Heilbronn, Max-Planck Str. 39, Heilbronn 74081, Germany; Hochschule Heilbronn, Heilbronn, Germany; Hochschule Heilbronn, Heilbronn, Germany

**Keywords:** colorectal neoplasms, cost-effectiveness analysis, disease model, health economics, liquid biopsy, Markov, medical, microsimulation, recurrence

## Abstract

**Background:**

Next-generation sequencing liquid biopsy (NGS-LB) for colorectal cancer (CRC) detection and surveillance remains an expensive technology as economies of scale have not yet been realized. Nevertheless, the cost of sequencing has decreased while sensitivity has increased, raising the question of whether cost-effectiveness (CE) has already been achieved from the perspective of European healthcare systems.

**Objectives:**

This health economic (HE) modeling study explores the CE of NGS-LB for CRC based on direct treatment costs compared to standard care without liquid biopsy in Spain, France, and Germany.

**Methods:**

A structured literature search was used to collect evidence from 2009 to 2020 on the stage-dependent quality of life (quality-adjusted life-years, QALY), efficacy, and total direct treatment costs (TDC) of NGS-LB. A decision-analytic Markov model was developed. Over the remaining lifetime, cumulative life expectancy (LE), TDC, and QALYs were calculated for 60-year-old men and women in CRC stage III with different assumed effects of NGS-LB of 1% or 3% on improved survival and reduced stage progression, respectively.

**Results:**

The use of NGS-LB increases LE by 0.19 years in Spanish men (France: 0.19 years, Germany: 0.13 years) and by 0.21 years in Spanish women (France: 0.21 years, Germany: 0.14 years), respectively. The 3% discounted cost per QALY gained was 35,571.95 € for Spanish men (France: 31,705.15 €, Germany: 37,537.68 €) and 35,435.71 € for Spanish women (France: 31,295.57 €, Germany: 38,137.08 €) in the scenario with 3% improved survival and reduced disease progression. Compared to the other two countries, Germany has by far the highest TDC, which can amount to >80k euros in the last treatment year.

**Conclusion:**

In this explorative HE modeling study, NGS-LB achieves generally accepted CE levels in CRC treatment from the health system perspective in three major European economies under assumptions of small improvements in cancer recurrence and survival. Confirmation of these findings through clinical trials is encouraged.

## Plain language summary

Is it worthwhile to use next generation liquid biopsy for cancer recurrence detection on patients with colorectal cancer?

Colon cancer is common. Worldwide, almost one million people die from it every year. Next Generation Sequencing Liquid Biopsy is a very sensitive technology for detecting cancer cells and their genetic information in the blood. Therefore, it is a good way to detect cancer and to detect early recurrence of a previously treated tumor. This test procedure is not yet used very often. Therefore, it is still expensive. Furthermore, there are still no studies that have demonstrated that and how liquid biopsy can aid doctors and patients after initial treatment. The research team of this study has developed an analytical model to investigate what performance liquid biopsy should have to demonstrate an affordable patient benefit in terms of quality of life, survival and cost per additional quality-adjusted life year gained. To do this, they studied the existing medical literature and many cost studies on colorectal cancer for the countries of Spain, France and Germany to feed their model. Then, they made different assumptions about the performance of liquid biopsy and did calculations. In the process, they also particularly examined the significance of specific influencing factors such as costs or disease progression in so-called sensitivity analyses. As a result, the authors found that there are large differences in treatment costs for colorectal cancer between the three countries Spain, France and Germany. Furthermore, even small improvements in the progression of cancer and the survival of cancer patients lead to the economic efficiency of liquid biopsy for the health care system. However, these are still thought experiments, so the research team of this study says that there should be further clinical trials to assess the impact of liquid biopsy on cancer progression and patient survival by using this technology. By this, one could confirm or contradict the authors’ educated assumptions and possibly pave a new way towards medical progress for people with colorectal cancer.

## Introduction

In 2020, colorectal cancer (CRC) was the third most common cancer type worldwide. With 1.9 million newly diagnosed cases and an estimated 935,173 deaths, it is the second deadliest cancer type.^1^ CRC can be categorized into four stages, which all have different probabilities to recur within 3 years – from about 5% in stage I up to 30% in stage III.^2^ Nonetheless, specialized treatment in higher disease stages increases the economic burden of CRC care.^3^

The current gold standard diagnostic methods to detect CRC are colonoscopy and fecal occult blood testing (FOBT). Unfortunately, these methods are unpleasant for patients and therefore non-adherence is an issue.^4^ Screening rates with colonoscopy are rather low in many countries and lower than with FOBT tests alone.^5^ Also their sensitivity only ranges from 19% in stages without symptoms to 51% when patients experience symptoms,^6^ making them insufficient for early CRC detection and cancer monitoring.^7^

In recent years, a new diagnostic method called liquid biopsy (LB) emerged, which shows a higher sensitivity in screening and recurrence detection.^6,7^ Its further development is called next-generation sequencing liquid biopsy (NGS-LB) which diagnoses CRC by detecting circulating genetic tumor information (ctDNA) in the blood. Multiple recent publications suggest that CRC recurrence is much more likely to occur where ctDNA is detectable through LB.^8–10^ There is potential for this technology to improve CRC care not only in detecting cancer but also by early detection of cancer recurrence.^11^ Suzuki *et al.*^12^ detected tumor recurrences on average 11.5 months earlier than radiological imaging, supported by further studies.^4,6^

The fourth edition of the *2019 Clinical Guideline for Genetic Testing in CRC Treatment* from the Japanese Society of Medical Oncology recommends the use of LB as a low-invasive and rapid diagnostic tool.^13^ NGS-LB is however to date not used widely in clinical settings nor recommended for general use in the 2022 guideline of the European Society for Medical Oncology for the screening and monitoring of treatments in cancers. Yet, the first specific recommendation has been made on ‘genotyping advanced cancer to guide therapies as an alternative to a tumor-guided strategy, especially in situations where tissue biopsies are suboptimal, or time is crucial’.^14^

Specific health economic (HE) studies in CRC care are still missing in Europe, and the question arises as to whether cost-effectiveness (CE) has already been achieved from the perspective of European healthcare systems.

Core European countries such as Spain, France, and Germany have heterogeneous healthcare systems but in common a strong pillar of public healthcare.^15^ At the same time, these countries generate high gross domestic products compared to the global community, which allows decision-makers to spend significant sums on their healthcare systems. The question is whether NGS-LB brings added value and economic use of limited budgets for these countries.

HE disease models are frequently used to answer such questions, and they have already been applied to NGS-LB topics in other cancer types.^16^ Computer simulations are needed because it is difficult to observe populations of cancer patients over the whole course of the disease in clinical trials.^17^ A sound HE evaluation requires that all relevant costs and consequences of a disease are observed and published to create fair comparison scenarios.^18^

This HE study investigates the CE of NGS-LB for CRC based on total direct treatment costs (TDC) compared to standard care without LB in Spain, France, and Germany. A HE disease model for CRC was developed based on scientific publications from a structured literature search on this disease, its associated costs, and the use of LB.

## Methods

This analysis follows the Consolidated Health Economic Evaluation Reporting Standards (CHEERS) published in 2022.^19^ The checklist has been made available as Supplemental Material.

In the first step, a structured literature review was performed for the years 2009–2020 to identify scientifically sound evidence that LB has added value in CRC treatments. This included answering the following research questions.

1. Is there a relevant additional benefit in the medical outcome categories? These include survival, quality-adjusted life-years (QALY), and tumor-free time between the occurrence of disease episodes, especially tumor recurrence, and the development of metastases.
2. Is there an additional economic benefit? This includes the costs of diagnostics, the monetary impact on medical treatment processes, and the avoidance of other expenses.
3. Are there established HE models that allow a plausible HE assessment? Are these available for the development of an HE model from the payer’s perspective?

**Figure 1.**
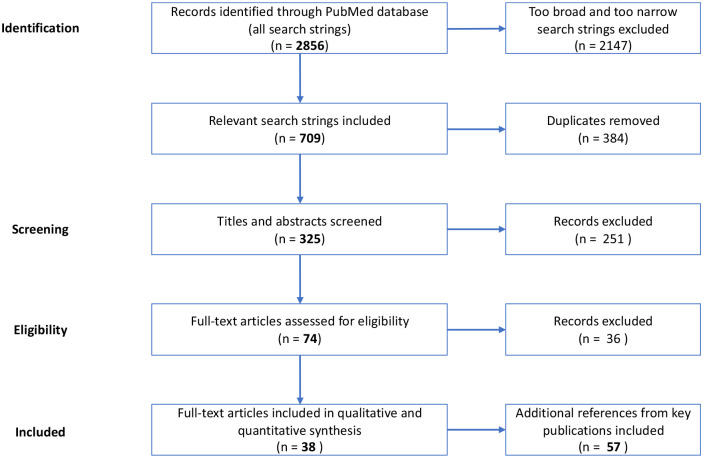
Flow diagram of the structured literature review.

In total, 2856 papers were identified by search strings in the PubMed service of the National Library of Medicine,^20^ leading after stepwise refinement to 38 relevant publications after screening of titles, abstracts, and full texts. The literature review results were then expanded by analyzing additional references from key publications, especially for the construction of the cost data set expanding the review to 57 full-text analyses. All literature was assessed by two independently working peers and consolidated where deviation in opinions was found (Figure 1).

An internal HE analysis plan was developed after the extensive literature review to define the elements of this HE study, the structure of the HE disease model, and the cost data set used in this study.

### Study population

This simulation analysis used a hypothetical patient cohort starting in CRC stage III with the age of 60 since age is one of the main risk factors of CRC.^21^ Therefore, no ethics vote was required for this study. Previous costs and treatments were not considered. Yet, model parameters and TDC were defined also for earlier cancer stages. We assume that all patients receive standard treatment throughout the simulation. The simulations were performed independently for men and women.

### Setting and location

The three European target countries Spain (ESP), France (FRA), and Germany (GER) have in common a mixture of a primarily strong public healthcare system that is supplemented to different amounts by private or voluntary health insurance and tax subsidies. Thus, representing a mix of the so-called Bismarck and the Beveridge health financing.^15^ Much of the reimbursement in the three countries is based on lump sums calculated by diagnosis-related groups.

### Comparators

This simulation uses in total seven main comparators. The first one is the ‘standard care’ comparator, which aims to simulate the situation as it is. As published in the literature, standard care includes colonoscopy or FOBT as a diagnostic measure and surgical removal with or without adjuvant chemotherapy for treatment according to the need.

All other six comparators are a copy of the first one but, additionally, use NGS-LB test kits in annual cycles to detect recurrence. They deviate among themselves by different assumptions on the medical effectiveness of NGS-LB. For this exploratory study, we assumed an improvement in:

- Survival by 0%, 1%, and 3%, respectively (MortSuccess).
- Decreased stage progression by 0%, 1%, and 3%, respectively (TxSuccess).

Men and women were modeled separately due to expected varying shares between both sexes over time due to different life expectancy (LE) of the sexes. All computations were then performed for the three target countries reflecting differences in the general mortality leading to a total of 36 comparative HE assessments against ‘standard care’ (3 countries ^★^ 2 sexes ^★^ 6 comparators against standard care).

All these presumed possible effects of better cancer recurrence detection could happen alone or in combination.

### HE perspective

This study adopts the HE perspective of the healthcare system of the three major European economies, Spain, France, and Germany, as described in respective publications.

### Time horizon

The model simulates the remaining lifetime of the individuals. Therefore, the model stops when the last patient enters one of the absorbing states ‘general mortality’, ‘CRC death’, or 60 years have passed.

### Discount rate

Both costs and consequences were discounted at 3%, following the suggestion of the German regulation.^22^

### Selection of outcomes

The medical benefit is quantified by two standard outcomes in cancer care and in HE studies: the cumulative LE and QALYs. They were calculated for all model comparators. These measures were selected, due to the assumption, that earlier detection of CRC leads to an earlier start of new or adjusted therapy, which increases the life expectancy (LE) and the quality of life.

For determining the economic benefit, the TDC is used for each comparator, respectively. Due to the literature-based cost assessment and the poor reporting quality, a further subdivision into cost categories was not possible.

For HE evaluations, the indicator’s total cost per life-year gained (C/LYG) and total cost per quality-adjusted life-year gained (C/QALYG) were calculated by the disease model in the comparative assessment of ‘standard care’ to the total of 36 possible scenarios. All of these parameters are used regularly in HE assessments.^23^

### Measurement of outcomes

To measure the HE outcome, the incremental cost-effectiveness ratio (ICER) is calculated for a willingness to pay (WTP) threshold of 23,500 € reflecting on the WTP threshold of 20,000 £ per QALY gained by the National Health Service (NHS) in England.^24^ The countries Spain, France, and Germany do not have their own WTP thresholds.^15^

### Valuation of outcomes

All data were taken as stated and summarized in the literature review and applied to a hypothetical cohort of 60-year-old men and women, respectively.

### Measurement and valuation of resources and costs

Costs for the integration of a next generation sequencing analyzer system as well as staff training were not considered. Costs for the additional NGS-LB are composed of costs of DNA extraction from blood samples, sequencing, other reagents, consumables as well as the NGS-LB test kit and are in line with published cost aggregations.^25^

### Currency, price data, and conversion

All costs are reported in Euros and were taken from relevant cost literature. The TDC for CRC care was divided into three main categories: initial, follow-up, and advanced costs (Table 1). The latter reflects the higher TDC of the last treatment year in cancer patients. Historical data were adjusted at rates of 4% for Spain and France and 3% for Germany reflecting national recommendations for the discounting of costs.^22^

**Table 1.** Total direct treatment costs for Spain, France, and Germany adapted to the reference year 2019.

Cost categories	Cancer stages	Spain	France	Germany
		Costs € (2019)	Costs € (2019)	Costs € (2019)
Initial costs (first year)	CRC stage I	16.795,80 €^3^	13.584,48 €^21,26^	3.819,31 €**
	CRC stage II	20.043,33 €^3^	17.488,52 €^21,26^	3.819,31 €^27^
	CRC stage III	21.451,55 €^3^	24.553,72 €^21,26^	37.552,13 €^27^
	CRC stage IV	2.161,49 €^3^	29.300,69 €^21,26^	101.655,77 €^27^
Follow-up costs (annual)	CRC stage I	2.308,54 €^3^	601,91 €**	3.084,29 €**
	CRC stage II	2.041,31 €^3^	601,91 €^21,26^	3.084,29 €^28^
	CRC stage III	1.478,37 €^3^	845,07 €^21,26^	3.084,29 €^28^
	CRC stage IV	1.478,37 €*	1.008,46 €^21,26^	3.084,29 €^28^
Advanced costs (last-year)	CRC stage I	6.980,60 €^3^	17.488,52 €^21,26^	80.890,87 €**
	CRC stage II	25.357,05 €^3^	24.553,72 €^21,26^	80.890,87 €^27^
	CRC stage III	40.802,11 €^3^	29.300,69 €^21,26^	80.892,14 €^27^
	CRC stage IV	40.283,29 €^3^	29.300,69 €^21,26^	80.690,72 €^27^

* /** assumption based on *previous or **following cancer state and respective reference. Costs for the CRC stages I and II are shown for transparency reasons but were not used in this study.

CRC, colorectal cancer.

The NGS-LB reimbursement amounts to 441,45 € per test according to the 2019 French reimbursement code N452 of the Le référentiel des actes innovants hors nomenclature (RIHN)^26^ representing a lump sum with an assumed 50% reimbursement rate for all services excluding blood collection and management costs of blood samples. This sum is in line with published reimbursement data in Euros for the Netherlands.^25^

### Rationale and description of the model

The CE analysis was based on a Markov model and Monte Carlo simulations, respectively, developed with the software TreeAge Pro Healthcare 2022 R1.1.^29^

**Figure 2.**
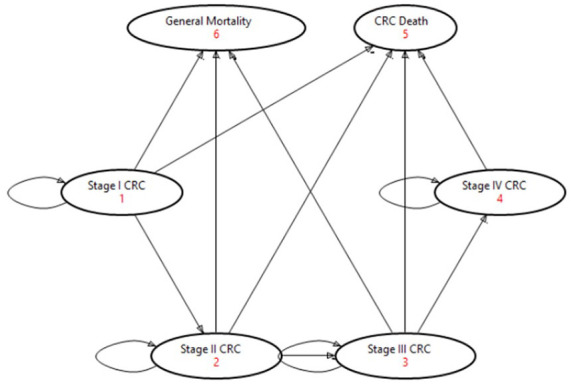
Simplified schematic of model states and transitions of disease progression in CRC. CRC, colorectal cancer.

This Markov-Monte-Carlo model consists of six states, of which two are absorbing states, as shown in Figure 2. This model structure includes CRC stage I–IV, the general mortality stage, and the CRC death stage. In the current literature, CRC stage jumps to earlier cancer stages are clinically documented, but due to a lack of evidence-based quantities, the model was simplified so that patients only recur in the next higher stage in line with other published disease models by Sharp *et al.*^30^ and Whyte *et al.*^31^ After successful therapy, patients stay in their current stage for regular follow-ups until they progress again due to cancer recurrence or die from general mortality or CRC mortality.

### Analytics and assumptions

The main assumptions in this study were that improved cancer surveillance leads to earlier detection of recurrence, which may have two fundamental implications:

First, by starting therapy earlier, patients can be kept in one stage of cancer (here initial stage III) longer. The impact of a small improvement of 1% or 3% was investigated.

Second, starting treatment earlier can reduce mortality. Here, the effects of a small improvement of 1% or 3% were investigated. These assumptions were realized by simple factor multiplications on the probabilities for cancer progression and mortality (last line of Table 2). All effects could happen alone or in combination with each other.

**Table 2.** Model parameters used for the computations.

Model parameters	Value and reference	Remarks
Start age (years)	60	Own assumption
Quality of life factor in CRC stage I	0.74^32^	
Quality of life factor in CRC stage II	0.74^33^	
Quality of life factor in CRC stage III	0.67^33^	
Quality of life factor in CRC stage IV	0.25^33^	
Annual probability of not having a recurrence after therapy for CRC stage I	0.983^2^	
Annual probability of not having a recurrence after therapy for CRC stage II	0.944^2^	
Annual probability of not having a recurrence after therapy for CRC stage III	0.887^2^	
Regular probability of dying from CRC stage I	0.015^34^	
Regular probability of dying from CRC stage II	0.039^35^	
Regular probability of dying from CRC stage III	0.078^35^	
Regular probability of dying from CRC stage IV	0.33^35^	
General mortality table for France, Spain and Germany	Table values (not shown)	General mortality tables based on annual age groups for males and females for the year 2019 by INE (Spain), INSED (France), and DESTATIS (Germany)
Probability of recurrence in CRC stage I	0.01^36^	
Probability of recurrence in CRC stage II	0.025^36^	
Probability of recurrence in CRC stage III	0.077^36^	
Discount rate events and costs	0%, 3%^22^	0% means undiscounted
A value whose probability is multiplied by the specific mortality and probability of recurrence of CRC in stages I–IV to simulate the effect of NGS-LB.	1/0.99/0.97	Own assumption

Parameters for the CRC stages I and II are shown for transparency reasons but were not used in this study.

CRC, colorectal cancer; NGS-LB, next-generation sequencing liquid biopsy.

The probabilities for cancer recurrence were taken from 3-year cumulated incidences by Qaderi *et al.*,^2^ for the stages I–III. The stage IV was assumed to be persistent.

Death rates from CRC were derived from the German Center on Cancer Registry Data including reported data until the year 2018.^34^ It was assumed that these rates apply to Spain and France as well.

For the use of NGS-LB, while lacking European medical guidelines on its utilization, it was assumed that one initial test was performed when starting the simulation and when switching from disease stages III–IV. In addition, annual tests were performed as part of the follow-up regime.

An implicit assumption of this study is that patients in stage III or stage IV cannot be healed, which represents a conservative assumption as the economic value of NGS-LB is systematically underrated. Table 2 summarizes the model parameters used for the computations of this study.

### Characterizing heterogeneity

As described, heterogeneity among patients is limited to age and sex. The heterogeneity of the results is taken into account by 48 independent calculations in a total of 36 comparison scenarios.

### Characterizing distributional effects

Due to the literature-based assessment, a sound estimation of data distributions was not possible. Own assumptions on data distributions were not made here and therefore data were taken as reported and no stochastic sensitivity analyses (SAs) were performed.

### Characterizing uncertainty

A test scenario identical to ‘standard care’ was designed including the costs for NGS-LB to allow for additional validation purposes. It was to calculate identical medical outcomes but with different costs and CE parameters. Through this procedure, the internal integrity of the model was cross-checked while applying scenario-specific changes and performing a total of 42 independent calculations.

The technical validation was performed using the built-in functionalities of the TreeAge software such as so-called roll-back analyses and cross-checks on probabilities above 100% among others.^29^

Internal validation was performed using one-way SAs by varying all independent cost and medical model variables by ±10% and recording the computations for the C/QALYG.

### Approach to engagement with patients and others affected by the study

Neither patients nor physicians were affected by this literature-based study. The conduct of the study had no impact on patients or other persons.

## Results

Of the 36 scenario analyses, those with the assumed NGS-LB effects of 3% reduction in stage progression with and without reduction in specific cancer mortality of 3% were found to be most effective. Therefore, these results are presented here (Table 3). All other calculations are presented in the result tables of the six models in the Supplemental Material. Furthermore, only the results discounted for costs and medical outcomes with an interest rate of 3% are presented following established HE standards.^23^ The results of the non-discounted calculations are similar and do not affect the interpretation of the study.

**Table 3.** Main health economic results for males and females (reference year 2019, discounted at 3%).

Strategy	Cost	Incr Cost	Eff Life-years	Incr LYrs	ICER (C/LYG)	QALY	Incr QALY	ICER (C/QALYG)
Female								
Germany								
Female – standard care	205,569.51 €		7.05			4.15		
Female – MortSuccess 0% – TxSuccess 3%	206,916.55 €	1,347.04 €	7.11	0.06	22,632.85 €	4.20	0.05	27,034.93 €
Female – MortSuccess 3% – TxSuccess 3%	211,461.75 €	4,545.19 €	7.25	0.14	32,145.93 €	4.27	0.12	38,137.08 €
France								
Female – standard care	86,046.26 €		7.14			4.20		
Female – MortSuccess 0% – TxSuccess 3%	88,156.30 €	2,110.04 €	7.20	0.06	33,902.79 €	4.25	0.05	40,864.37 €
Female – MortSuccess 3% – TxSuccess 3%	89,905.05 €	3,858.79 €	7.35	0.21	18,518.02 €	4.32	0.12	31,295.57 €
Spain								
Female – standard care	108,014.02 €		7.18			4.22		
Female – MortSuccess 0% – TxSuccess 3%	110,004.75 €	1,990.73 €	7.24	0.06	31,594.80 €	4.27	0.05	38,143.01 €
Female – MortSuccess 3% – TxSuccess 3%	112,429.27 €	4,415.25 €	7.39	0.21	20,956.46 €	4.35	0.12	35,435.71 €
Male								
Germany								
Male – standard care	196,960.10 €		6.76			3.99		
Male – MortSuccess 0% – TxSuccess 3%	198,166.67 €	1,206.58 €	6.81	0.05	22,776.88 €	4.03	0.05	26,655.31 €
Male – MortSuccess 3% – TxSuccess 3%	202,245.09 €	4,078.41 €	6.94	0.13	31,600.31 €	4.09	0.11	37,537.68 €
France								
Male – standard care	82,746.33 €		6.83			4.03		
Male – MortSuccess 0% – TxSuccess 3%	84,730.03 €	1,983.70 €	6.88	0.06	36,029.60 €	4.07	0.05	42,512.71 €
Male – MortSuccess 3% – TxSuccess 3%	86,292.16 €	3,545.83 €	7.02	0.19	18,878.78 €	4.14	0.11	31,705.15 €
Spain								
Male – standard care	103,672.52 €		6.89			4.06		
Male – MortSuccess 0% – TxSuccess 3%	105,533.55 €	1,861.03 €	6.95	0.06	33,260.28 €	4.11	0.05	39,310.18 €
Male – MortSuccess 3% – TxSuccess 3%	107,707.93 €	4,035.41 €	7.08	0.19	21,169.03 €	4.17	0.11	35,571.95 €

C/LYG, cost per life-year gained; C/QALYG, cost per quality-adjusted life-year gained; ICER, incremental cost-effectiveness ratio; QALY, quality-adjusted life years.

### Medical outcomes

As summarized in Table 3, females aged 60 with cancer stage III and under standard care live for another 7.05 years in Germany, up to 7.18 years in Spain with French women being close to them (7.14 years). With the described assumptions of this study, the model estimates an increase of 0.14 life-years in German women compared to 0.21 years in France and Spain. The QALY results remain below these levels with a spread of 4.15 QALYs in German women under standard care to a maximum of 4.35 QALYS in Spanish women with NGS-LB, followed closely by French women with NGS-LB at 4.32 QALYs.

As expected, men show lower outcomes with around 0.3 life-years shorter survival and 0.2 lower QALYs.

### Economic outcomes

With 211.461,75 €, the HE model estimates the highest TDC for German women under NGS-LB, which is more than twice the TDC of French women and almost twice the cost of Spanish women. Similar results can be noted for men.

### HE outcomes

Against the chosen WTP of 23,500 € C/QALYG, none of the calculated scenarios stays below this threshold, but some of them come close. If one chooses a WTP of 50,000 € C/QALYG, all scenario analyses will stay below this level ranging from 26,655.31 € C/QALYG in German men with a 3% postponement of stage progression to 42,512.71 € C/QALYG in French men with the same medical effect of NGS-LB. All scenarios stay below the U.S. American WTP threshold of $ 50,000 C/LYG.^23^

#### Effect of uncertainty

Due to the relatively high age of the patient cohort, that is, 60 years, the general mortality has a measurable influence on survival. Females live slightly longer than men. The 50% survival threshold is recorded at 18–19 years from simulation start in men and slightly more at 20 years in women, respectively. Spanish men and women have got a slightly higher LE than patients from France and Germany, but these effects are small (Table 3). An additional table with computed survival data has been attached as Supplemental Material to provide longitudinal developments over time to the interested reader.

#### Results of the SAs, study parameters

Numerous one-way SAs were computed to assess the impact of independent model parameters and variables on the CE outcomes.

**Figure 3.**
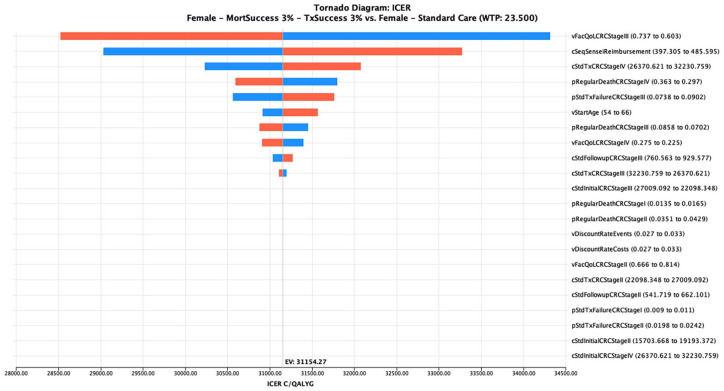
Tornado diagram on cost per QALY gained in French women. QALY, quality-adjusted life years.

Naturally, the highest impact of NGS-LB on CE is to be expected in scenario analyses of the maximum NGS-LB effect of 3% MortSuccess plus 3% TxSuccess in the cheapest of the three healthcare systems for the patients with the longest LE. Thus, Figure 3 illustrates the tornado diagram resulting from the SA for French women for the C/QALYG. Here, one recognizes that the three most influencing variables are quality-of-life while being in cancer stage III with a 9.2% change in this ICER, followed by the reimbursement costs of NGS-LB with a 6.8% change followed by the TDC of cancer treatment in stage IV with a 2.9% change.

The asymmetry of the influencing factor ‘start age’ mirrors the exponential increase of age-dependent mortality in the general mortality tables.

In summary, one can conclude that the CE results are sensitive to the main medical outcome parameter QALY and to a lesser extent the NGS-LB costs and treatment costs. Nevertheless, all forced swings of the results remain below 10% change from baseline.

## Discussion

### Current knowledge

Recently, an important non-inferiority study by Tie *et al.*^37^ demonstrated that NGS-LB is beneficial in clinical decision-making regarding adjuvant chemotherapy after initial tumor surgery in stage II colon cancer patients. Though not intended by this lively discussed study,^33,35,38^ it surely realizes a cost-saving using the comparatively cheap NGS-LB test to avoid more costly adjuvant chemotherapy and proved no worse outcome in participating patients. The weakness of this approach is that no progress is made in cancer therapy and ultimately the patients bear the risk. Why, for example, should one expect the same relevant patient outcome when the decision to forego adjuvant chemotherapy is made by less specialized clinical centers than in this cited study? The unmet need for better evidence on the clinical effectiveness of NGS-LB in CRC treatment monitoring and related CE persists.

Only a few publications dealt with HE modeling for CRC, and no publication was found at the time of conducting this research that covers the evaluation of the cost–benefit of LB for CRC recurrence detection. Extensive literature research identified few HE publications with a different focus than the presented work. Barré *et al.* compared different screening methods, where blood-based testing was found to be not cost-effective but showed the highest increase of QALY. They concluded that blood-based testing could be cost-effective in high-risk CRC cohorts.^21^ By contrast, Seo and Cairns reported that cancer biomarkers for targeted therapies in metastatic CRC were mostly found to be cost-effective or improved the CE of their targeted therapies. While the companion biomarkers reduced the costs, the savings were not sufficient to make the therapy overall cost-effective.^36^ This evidence of other working groups does not fit exactly with our analysis, but it is in line with our efficiency results.

### Study findings

When evaluating CE in this modeling study, the WTP threshold plays an important role in measuring success in this study. According to the HE analysis plan of this study, the English NHS threshold was applied, as a result, the WTP thresholds lie above €23,500/QALYG, and no CE is achieved. This is a negative result. However, it should be noted that the NHS uses one of the lowest thresholds in developed healthcare systems. By contrast, if the thresholds commonly used in other countries are used, NGS-LB for CRC is efficient. Neumann and Kim^39^ have investigated such alternative WTP thresholds over three decades and reported that for some time, most HE studies have calculated WTP thresholds in the range of $ 50,000–$ 100,000 for C/QALYG. It is therefore fair to say that NGS-LB has already reached the threshold for CE in many, if not most developed healthcare systems. This conclusion does not yet consider scaling effects to make this technology cheaper through widespread use and more cost-effective production at scale. Spain, France, and Germany do not use WTP thresholds at all. The assessment of the study results presented here thus remains a judgment call in these healthcare markets.

In this study, small clinical improvements of 1% or 3% in survival and or disease progression were assumed, and encouraging the CE results were calculated. With improved clinical effects, these results would be highly favorable for NGS-LB. This is countered by a current lack of study-based data. Is a 5% improvement in survival or a reduction in disease progression feasible by NGS-LB monitoring for early recurrence detection? To date, this is not validated knowledge, but this could be relevant for CRC patients. Clinicians are encouraged to consider conducting longitudinal prospective randomized clinical trials investigating cancer surveillance and early detection of cancer recurrence using NGS-LB. The HE analysis model presented here is open to the scientific community to replicate the results of this study, if appropriate.

One would expect that in all scenario analyses the maximum NGS-LB effect on survival in combination with reduced stage progression would show the best ICER. This is the case in most but not all analyses. It appears that the reduction in stage progression plays a more important role than the reduction in cancer mortality. This is shown, for example, in the ICER in women from Germany (Table 3). However, this is a logical fallacy. Based on our assumptions, in this HE evaluation, improved medical care leads to longer survival, which, in turn, is associated with longer payment duration from the payers’ perspective. The ICER then represents a mixed calculation. Furthermore, a reduced stage progression means that the lower mortality probability of stage III compared to stage IV also contributes to reduced mortality. The two target parameters for the effectiveness of NGS-LB are possible outcomes of improved disease monitoring through NGS-LB, but they are not clearly independent of each other. Within certain limits, these shifts in reported results can therefore occur.

### Limitations

During the model development process, several principal decisions were to be made regarding the cost of cancer care. In this study, the treatment costs for each stage I–IV were divided into initial costs including diagnosis and first treatment, annual follow-up costs, and advanced costs for the last treatment year. This determination led to the exclusion of other valuable cost literature. For instance, the detailed cost report by Com-Ruelle *et al.*^40^ for France could not be used here. Also, it would have been valuable to have a realistic distribution of cancer patients across the disease states as published, for example, by Cottet *et al.*^41^ (i.e. 17% stage I, 31% stage II, 22% stage III, and 30% stage IV), but it was not possible to find comparable data for all three countries. Nevertheless, the CRC disease model can easily be adapted to such improved scenario analyses. We limited this study to three important European health markets. However, interesting cost data and CE analyses have been published for other European healthcare markets such as Italy.^42^ Specifically, the publication by Pisapia *et al.*^43^ showing that the cost of performing NGS-LB is significantly higher in Italy raises the legitimate question of whether the reimbursement codes we used in our CE study reflect the true cost of clinical laboratories for LB or merely indicate systematic underfunding for this technology.

The development of NGS-LB is very dynamic. One observes an increasingly improved sensitivity of the test kits in the last years. Recently, this development has led to the justified conclusion that NGS-LB should be used for therapy decisions on the necessity of adjuvant chemotherapy.^14^ Unfortunately, the disease model used here cannot process the increased sensitivity of NGS-LB as a parameter. There is a need here for further development of the Markov model used based on better knowledge than hitherto.

For the cost of NGS-LB, a reimbursement rate of 50% on the French reimbursement code RIHN/N452 of 882,90 € based on personal communication of active researchers in France was assumed,^26^ while published data on routine reimbursement were not available at the time this study was conducted. This assumption was part of the SAs, which did not show a dominant influence on CE. This study does not reflect on cost savings that are induced by the increased use of NGS-LB which likely will induce reduced manufacturing prices through the mechanisms of an economy of scales and increased competition. It also does not look at the likely improvements in the automation of the laboratory procedure through standardized, instrument-compatible test kits that achieve robust results without the need for highly qualified and thus expensive knowledge.

In this study, the patient characteristics were restricted to an initial age of 60 years in CRC stage III, which is comparatively late in the course of the disease. This decision had been previously specified in the internal HE analysis plan. It follows from the logic of this modeling approach that CE is improved if NGS-LB surveillance is started at earlier cancer stages, especially stage II. Indeed, most of the seven scenarios of this study would then be highly cost-effective. However, after reviewing the relevant literature, the dominant opinion was that the clinical evidence is too uncertain to justify this decision against the background of the patchy clinical evidence. Progression from CRC stage II to CRC stage IV was reported in clinical studies, but a reasonable probability for stage jumps was not reported.^44^ The event happens, but to what extent exactly? And are there country-specific differences? Overall, the analysis presented here must therefore be judged as very conservative due to the limitation on CRC stages III and IV only. In fact, the decision to start monitoring late systematically underestimates the true benefits of NGS-LB in this aspect. Following the principle of iterative HE assessments,^18^ this analysis could easily be repeated when better evidence is available. The Markov model is prepared to deal with this specific and important clinical question.

External validation against published results from literature reports (e.g. medical studies, registry data, longitudinal studies) was not possible here, as most of the available published evidence had to be used for the model parameters themselves. It was not possible to find adequate literature not used for the model construction for all three target countries. Therefore, the validation is focusing on internal validation and specifically on SAs.

The high-cost differences between the three countries are mainly due to the high German TDC reported by Ladabaum and Mannalithara^27^ and Haug *et al.*^28^ In principle, this study does not serve to compare therapy costs for CRC between countries, as published cost data were used, and the healthcare systems are heterogeneous in their care and financing structures. The publications used did not present the details in a manner that would allow direct comparability. The cost data used are therefore not for comparing countries, but exclusively to calculate efficiency results of cancer monitoring. It is nevertheless remarkable that the very high TDC in Germany is not accompanied by dramatically different CE indicators. The reason for this finding is the comparatively low price for NGS-LB in relation to the high TDC in the CRC stages III and IV.

Another important limitation is the use of many assumptions in this study, which, in turn, affect quite a large set of outcome measures (Table 3). It is an impossibility to present all results in a printed document. For this reason, only the most important and crucial scenario results for the research questions are presented here. The interested reader is referred to the Supplemental Material, which contains fully functional models and all calculations that did not make it into the manuscript.

This study covers the period before the COVID-19 pandemic, which started in Europe in early 2020, and before the Ukrainian–Russian war, which started in 2022. The disadvantage of this time period is that the data used are comparatively old. The advantage is that the assessment of NGS-LB here is based on a largely stable situation for the European healthcare systems. Since then, the financing situation has drifted further apart due to different inflation rates and state aid for the healthcare systems in Europe, which would make it difficult to interpret more recent analyses.

Regarding the discounting of costs and consequences, the rate of 3% was based on German guidelines. Other interest rates could have been used in better consideration of the healthcare systems in Spain and France. Nevertheless, a largely consistent comparison scenario is needed for this study. Furthermore, the SA demonstrates that no relevant distortions are to be expected from the choice of an interest rate.

### Generalizability

This is an exploratory HE modeling study and it is driven by the main question: ‘What if NGS-LB showed a defined magnitude of medical benefit?’, while exactly this evidence is still lacking. All results should therefore be interpreted with caution. The strength of this study is the use of an easily adaptable disease model to test certain hypotheses (here: CE compared to a WTP threshold in the examples of Spain, France, and Germany) and their relevance for important healthcare system decision criteria in Europe. The advantage is that new findings can be easily integrated, and the calculations can be repeated often, as well as the robustness of the original hypotheses can be validated.

New diagnostic and therapeutic procedures can never reveal all the benefits and risks at the beginning of widespread use, which is why the repeated use of technology assessment is recommended by notified bodies and experts.^18^ The exploratory study presented here provides reasonable evidence that the application of NGS-LB can already be assessed as cost-effective in many countries today, that is before widespread use and economies of scale are realized. As robust studies are lacking so far, it is recommended that prospective clinical studies with NGS-LB be conducted for the early detection of cancer recurrence. Two main objectives should be pursued from a HE perspective. First, better clinical data on the costs, the use, and the effects of NGS-LB as a valuable input to disease models, and second, clinical scenarios and publicly available data that support better external validation of the computed results of HE models and studies in the future. A HE evaluation should then be repeated to confirm or contradict the findings of this study.

## Conclusion

In this explorative HE modeling study, NGS-LB achieves generally accepted levels of CE in the CRC indication from the perspective of healthcare systems in three major European economies under assumptions of small improvements in cancer recurrence and survival. Confirmation of these findings through clinical trials is encouraged.

## Declarations

**Ethics approval and consent to participate:** Not applicable.

**Consent for publication:** Not applicable.

**Availability of data and materials:** The software TreeAge Healthcare Pro was used to program the disease models in this study. This highly specialized simulation software may not be transparent to readers of this publication. As the software allows a functional Excel export, the authors make six models for Spain, France, and Germany, both for males and females, respectively, available as Supplemental Materials. These models include the described seven comparators, model parameter values, and main references.
